# Tackling Performance Challenges in Organic Photovoltaics: An Overview about Compatibilizers

**DOI:** 10.3390/molecules25092200

**Published:** 2020-05-08

**Authors:** Aurelio Bonasera, Giuliana Giuliano, Giuseppe Arrabito, Bruno Pignataro

**Affiliations:** 1 Department of Physics and Chemistry-Emilio Segrè, University of Palermo, viale delle Scienze, bdg. 17, 90128 Palermo, Italy; giuliana.giuliano@studium.unict.it (G.G.); giuseppedomenico.arrabito@unipa.it (G.A.); 2 INSTM-Palermo Research Unit, viale delle Scienze, bdg. 17, 90128 Palermo, Italy

**Keywords:** additives, compatibilizers, bulk heterojunction, donor/acceptor interface, mixing interfaces, morphology modulators, organic photovoltaics

## Abstract

Organic Photovoltaics (OPVs) based on Bulk Heterojunction (BHJ) blends are a mature technology. Having started their intensive development two decades ago, their low cost, processability and flexibility rapidly funneled the interest of the scientific community, searching for new solutions to expand solar photovoltaics market and promote sustainable development. However, their robust implementation is hampered by some issues, concerning the choice of the donor/acceptor materials, the device thermal/photo-stability, and, last but not least, their morphology. Indeed, the morphological profile of BHJs has a strong impact over charge generation, collection, and recombination processes; control over nano/microstructural morphology would be desirable, aiming at finely tuning the device performance and overcoming those previously mentioned critical issues. The employ of compatibilizers has emerged as a promising, economically sustainable, and widely applicable approach for the donor/acceptor interface (D/A-I) optimization. Thus, improvements in the global performance of the devices can be achieved without making use of more complex architectures. Even though several materials have been deeply documented and reported as effective compatibilizing agents, scientific reports are quite fragmentary. Here we would like to offer a panoramic overview of the literature on compatibilizers, focusing on the progression documented in the last decade.

## 1. Introduction

Nowadays, third-generation Photovoltaics (PVs), including Organic Solar Cells (OSCs) [1,2,3], Dye-Sensitized Solar Cells (DSSCs) [4,5,6], and Perovskite Solar Cells (PSCs) [7,8,9], are making inroads, showing great potential to overcome some of the limitations of today’s applied PV technologies, and opening the door to novel, unconventional PV applications. In particular, the exponential growth in the number of investigations on the topic of Organic Photovoltaics (OPVs) [10,11] has produced a tangible evolution of these promising devices [12]. Increasing attention over OPVs is granted by a number of appealing/beneficial factors, comprising low-cost production investments [13,14], solution processability [15,16], compatibility with large-scale printing approaches [17,18], and established chemical pathways for organic synthesis and molecular engineering [19,20,21,22,23,24,25]. Moreover, the employ of organic materials guarantees the economically and environmentally sustainable life-cycle of the device [26,27]; this feature is particularly attractive if we consider that today’s public opinion pays more attention to the full-recyclability of the device, in order to meet the principles of circular economy and to prevent further threats to the environment and ecosystems. The concept of sustainable economic development relies on the employment of alternative protocols that minimize the environmental impact, following the principles of green chemistry. The limited employ of harmful chemicals is one of the possible approaches [28], as well as the utilization of ecofriendly biomaterials, such as bioinspired curcuminoid derivatives as molecular donors [29], or nanocellulose and nanochitin membranes in dye solar cells [30]. In the context of OSCs, progress in this direction has been obtained by using metal-mediated cross-coupling reactions that minimize organotin waste, using polycondensation reactions that evolve water as a by-product and even more interestingly, using natural-precursors-derived materials [31]. The adoption of sustainable life cycles is an aspect of particular importance, especially for natural sources carbon-based materials, which are at the base of relevant energy and electronics-based applications [32].

Additional beneficial features of OPVs comprise mechanical flexibility [3,33], lightness (around 500 g/m^2^) [24], and semitransparency [3]. These unique properties allow for the exploration of application fields that cannot be targeted by using conventional silicon photovoltaics [34]. One remarkable example is represented by building-integrated photovoltaics that combine lightness and transparency, along with sun protection and energy generation. Space exploration [35,36] is another cutting-edge application field which will be greatly benefitted by OSCs, for a series of important reasons: (i) their high processability is of great value in spaceships, where limited stocks of raw materials and minimum equipment are available to the crew members [37]; (ii) their versatility makes OSC simple to integrate as energy-source components for a number of apparatuses, ranging from diagnostic networks to propulsion systems [38]; (iii) the possibility to couple organic solar cells with the last-generation storage batteries [39] will guarantee a prolonged autonomy for the exploration missions directed at the outermost areas of our solar system.

OPVs success lies in their conceptual simplicity: They can be described as solid-state semiconductor devices acting as a diode in the dark, and, under illumination, they are able to convert incident light directly into electricity through the photovoltaic effect [40]. Unlike the conventional wafer-based PV devices, OPV cells are built from thin films (typically 100 nm) of organic semiconductors, including polymers and small molecules, which can absorb light in the UV/visible region and transport electric current, owing to their electronic delocalization. The photovoltaic conversion mechanism can be explained as follows: The photoactive material is excited by means of light, generating Frenkel excitons (tightly bound electron–hole pairs); free electrons and holes are then produced by exciton dissociation, which is induced by an interface across which the chemical potential of electrons decreases, and transported to the opposite terminals of the device [41]. If an electrical load is connected to the device between the front and back contacts, charge carriers will complete the circuit through this load and perform work. In the early days, the photoactive layer consisted of a thin film of a single organic material with light absorption capability deposited in between contacts [42]. However, low-power conversion efficiency (PCE) values were registered (< 0.1%) due to weak electric fields generated at the electrodes, unable to break excitons into pairs of free charge carriers efficiently. These devices, although pioneering, are confined to the pages of history books.

Although scientific reports describe geometries where three layers made of different materials are deposited one onto the other [43,44], current investigations mainly explore bi-layer OPVs, also called binary OPVs, where an electron donor (D) and an electron acceptor (A) material are deposited. The photon-absorbing material is usually referred to as the donor, whereas the acceptor is the partner material collecting electrons [45]. Here, the device typically comprises an electron- or hole-blocking layer on top of a conductive transparent glass or plastic (which allows photons to make a breach to the deeper layers of the device), then the electron D/A layers, a hole or electron blocking layer, and a metal reflective electrode deposited on top. Ordering in the D/A layers and the blocking layers is a consequence of the specific geometry of the device that can be defined as “regular” or “inverted” [46], depending on the arrangement of the electrodes and deriving from the nature of the employed materials and processing steps.

Initially, bi-layer devices featured planar heterojunctions between D- and A-layers [47]. The electric field generated at the junction promoted the separation and diffusion of holes and electrons to their specific collecting electrodes, but this geometry still afforded poor PCE values, mainly due to the limited exciton diffusion length [48]. The latter has been estimated in a few tens of nanometers, which means that only excitons generated within a short distance from the D/A interface have the possibility of dissociating into free electrons and holes before relaxing to the ground state [49]. Considering this, it was realized that, in order to prevent de-excitation and promote charge extraction, the interface area should be maximized, while at the same time ensuring the presence of percolated conduction pathways for charge carriers to the collecting electrodes. To this aim, the successful diffusion of Bulk Heterojunction (BHJ) geometries represents a cornerstone in the field [50,51]. Here, D and A are usually prepared as a blend, then cast as a mixture, and finally allowed to phase-separate. This approach guarantees an intimate mixing of the D/A components in a bulk volume, with average domain sizes in the order of tens of nanometers, so that the D/A interface locates within a distance less than the exciton diffusion length from each absorbing site. In this way, the exciton decay is reduced to a large extent, resulting in an appreciable enhancement of the device efficiency.

Although OPVs are very promising, some intrinsic limits of this technology should be evidenced [52]; PCE is still lower compared to old semiconductor-based products, and numbers are comparable only in the best cases [53]. The limitations with respect to OPV efficiency stem from the short diffusion length of excitons, as well as from the insufficient absorption of photoactive layers whose thickness is in the range of 100 nm (the low charge mobility of organic semiconductors obliges the active layer thickness to stay low), resulting in a low external quantum efficiency (EQE). However, it is worth mentioning that numbers are rapidly increasing, making statements susceptible to revision in the near future [54]. In particular, tandem Organic Solar Cells (OSCs) have recently achieved impressive performances, offering a viable approach to maximize the EQE: From the last record of 2013, claiming an encouraging 10% [55], the work of Chen et al. made its way to the podium of the top performances ever reported, with an extraordinary 17.3% [56]. The gap has been drastically reduced, compared to inorganic solar cells; nevertheless, it is mandatory to mention that silicon-based PVs have recently overcome the barrier of 26% PCE [57], and more sophisticated solar concentrators have been proved to possess the potential to exceed 50% efficiency [58]. The above results are a clear demonstration of the tremendous improvements obtained by the researchers, nevertheless there are still some important hurdles to be tackled before OPVs’ efficiency can really become competitive. Another significant weakness for the present OPV technology is the stability and relatively short lifespan of the devices [2,59]. Environmental stressors such as oxygen, moisture, heat, and UV radiation can induce significant degradation effects in OSCs. Strategies such as encapsulation can mitigate these parasite processes through protection from external stimuli [60] but cannot shield from intrinsic phenomena, such as interdiffusion between constituent materials and temporal changes in the nanoscale morphology of the active layer. Inherent instability can be tackled in some ways via careful component choice and/or through morphology control strategies [61]. However, it is clear that further research is needed to prolong the operational lifetime of OPVs through a deeper understanding of degradation mechanisms.

After these few lines, the first question arising in the reader’s mind should be as follows: How can OPVs be further optimized? Improvement of OSCs performance, both in terms of efficiency and stability, cannot leave aside an in-depth understanding of all the parameters influencing the final output of the devices: Materials development, morphology, device physics and structure, etc. are all intimately interconnected, and their separate analysis, although interesting, is highly time-demanding. For the purpose of this review, we can simplify and organize our considerations around three basic aspects, as follows.

### 1.1. The Photoactive Materials of Choice-Structural Parameters and Molecular Design

Organic photovoltaics are made up of a combination of donor and acceptor organic molecules [62], nanostructured materials [63], and/or polymer chains [62,64]. In this context, it is important to choose chemical structures able to absorb photons from the incoming sunlight, thus promoting electrons to an excited state and generating excitons. Photoactive materials are usually conjugated polymers, where a wide system of π-electrons delocalizes the electronic density along the organic scaffold. Delocalization corresponds to lower energy for promoting electrons from the molecule’s highest occupied molecular orbital (HOMO) to the lowest unoccupied molecular orbital (LUMO), denoted by a π–π* transition. The materials of choice should possess a HOMO-LUMO energy gap compatible with the energy brought by photons in the near UV/visible/near IR spectral window, so that solar light can kick off the starting event, to produce excitons. Even if we mentioned conjugated polymers, the discussion should be extended to a wide collection of chromophores, which have been successfully tested; in particular, chromophores with small aromatic cores (naphthalenes [65], pyrenes [66], and perylenes [67]) are typical examples of discrete molecules that are easy to handle and precisely modify (unlike polymers), and they are able to create ordered nanostructures that resemble polymer chains (columns, stacks of lamellae), due to π–π stacking interactions. Some relevant efforts have focused on the engineering of π-conjugated macromolecules by direct arylation strategies for BHJs [68], fluoro-functionalized quinoxaline-based polymers to be used as low-band gap acceptors [69], and finally perylene diimides as n-type organic molecules usable as non-fullerene acceptors in organic photovoltaics [70]. Generally speaking, strong π–π interactions are desirable, since they allow for the propagation of excitons and/or charge carriers through relatively long distance (like in a polymer chain), although there is not a physical network of bonded atoms. In this regard, several comprehensive reviews are greatly helpful to reveal design guidelines for molecular construction and chemical modification of π-conjugated materials toward higher-efficiency OPVs [71,72,73,74,75]. Features such as strong absorption, low HOMO-LUMO energy gap, high charge mobility, good film quality, and proper miscibility with the other blend component, are highly desirable for further increasing the device’s performance. It is worth mentioning another interesting alternative approach represented by the employ of hierarchical multicomponent metamaterials assembly nanostructures; in the literature, several examples are listed, such as layer-by-layer assemblies of silver nanowires [76] or TiO_2_ nanoclusters [77] to model photovoltaics devices morphology. These materials belong to the world of inorganic chemistry in most of the cases, but they can be usefully combined with organic partner(s) to form interesting hybrid architectures (for a as deeper analysis of the topic, we warmly recommend the recent review from Levchenko et al.) [78].

### 1.2. The Device Architecture, Energetic Considerations, and Interface Engineering

OPV technology involves the sequential deposition of conductor, semiconductor, and/or non-conductor materials in the form of thin films, to give a resultant device architecture that can be regular or inverted, depending on its polarity, as previously described. A careful selection of materials and interfaces and a proper alignment of the energy levels is crucial to achieve high-voltage outputs and assist directional charge extraction. In particular, engineering the interface between D/A layers is of great importance, since the charge separation process occurs in this area; the interface should generate a strong electric field and provide the necessary driving force to overcome the exciton binding energy, reducing the possibility for electrons and holes to match again as a geminate pair. More specifically, suitable energy offsets (at least 0.3 eV) between donor and acceptor, and proper matching of their HOMO-LUMO energy levels, are required to ensure efficient exciton dissociation and minimize carrier recombination [79]. In addition, the work functions of the electrodes must be carefully selected to provide selective contacts for charge carriers. The formation of a quasi-ohmic contact, in contrast to a Schottky contact, is commonly preferred to alleviate the interfacial energy barriers. Indium tin oxide (ITO; work function, WF = 4.7 eV) [80] is typically used as the transparent bottom electrode, acting as an anode for the collection of holes in conventional devices, or serving as a cathode for the collection of electrons in the inverted geometry. Concerning the metal-top electrode, to meet the energy match, a low (high) WF metal is usually required as a cathode (anode) in a regular (inverted) architecture. Additional measures, such as interfacial buffer layers and doping strategies, are continuously being developed, to reduce interfacial barriers and facilitate charge collection (toward higher PCEs), as well as to prohibit physical phenomena and/or chemical reactions at the interfaces (toward enhanced stability) [81].

### 1.3. The Donor/Acceptor Interface (D/A-I) – Morphological Properties and Nanoscale Evolution

**Figure 1:**
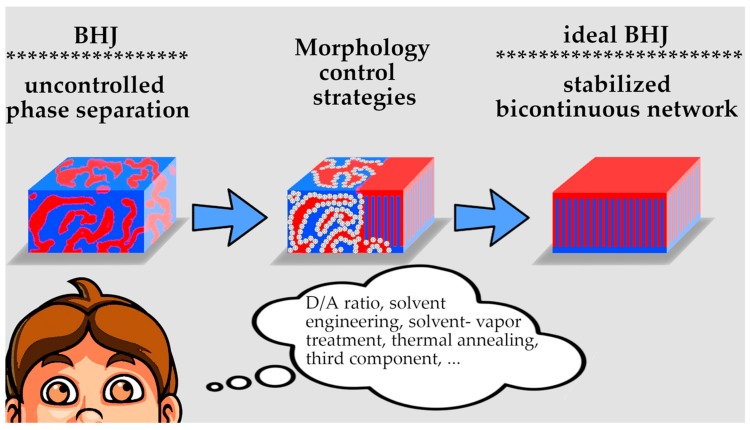
Pictorial representation of a bulk heterojunction. As suggested by Tommy, several strategies can be adopted, aiming at controlling the morphological profile at the microscale nanoscale and bringing the system closer to the ideal case.

The third basic aspect to be considered concerns the morphology and stability of the D/A heterojunction [82,83]. The photoactive layer is the key component of OSCs, in which there are generally two or three phases (the pure donor phase, the pure acceptor phase, and the D/A amorphous intermixed phase), depending on the degree of miscibility between donor and acceptor [84]. The phase-separated morphology of the D/A heterojunction is known to be critical for both the generation and extraction of charge in OPVs, with the lack of optimal phase separation being often responsible for poor device performances [85,86]. When planar junctions were mentioned, it quickly appeared how limiting such geometry could be; it does not allow for a maximization of the interacting interface, nor is helpful when considering the limited exciton diffusion length. Conversely, BHJs permit to increase the interfacial area and reduce the dimension of the isles produced by each phase, contributing to the efficient charge extraction. However, the production of a BHJ cannot be precisely ruled: After mixing donor and acceptor in a single solution and letting them phase-separate during film formation, a thermodynamically driven process emerges, where some isolated microphases (from both D- and A-materials) grow distantly from each other and surrounded by bulk mixture [87], thus making the photogenerated charge carriers unable to move to the external electrodes. The ideal morphology for BHJ in OPVs can be visualized as a bi-continuous interpenetrating network, whose interface resembles a herringbone pattern [88], as pictorially described in Figure 1. In this way, D/A-I is maximized for the efficient photocurrent generation, and, at the same time, the interdigitating portions from each phase guarantee rapid flow of electrons and holes to the corresponding electrodes, and then to the external circuit, without discontinuities. Engineering such an ideal interface is extremely intriguing for scientists, but challenging, as well. To be noticed, the D/A-I is not a physical object, meaning a specific material or a confined compartment of the device, but a precise morphological element produced neither by the donor nor by the acceptor layer, but by a complex sum of their interactions, which makes it very difficult to govern. Moreover, another outstanding issue is that the organic blends resulting from the BHJ approach often correspond to kinetically frozen states which have not reached thermodynamic equilibrium [89]. Not surprisingly, many cases of performance degradation due to nanoscale evolution of the BHJ morphology during device operation have been reported in the literature [90,91,92,93]. As a result, significant interest rises for strategies that can improve the performance of the devices through rational control and stabilization of the morphologies [61].

Based on the above classification, we can distinguish three main lines of the current OPV research: molecular design, interface engineering, and morphology control. Despite the astonishing progresses achieved in the last years, through innovative molecular design and interface engineering strategies, it is widely believed that optimized BHJ morphologies and nanoscale control hold the key to the design of commercially viable OSCs [94,95,96,97,98]. The controlled formation of a fine BHJ nanostructure that resembles the ideal one and that does not evolve with time is a key requirement for an efficient and stable OPV-technology. D- and A-materials should be judiciously chosen for this purpose, but there are several other parameters influencing the topology of the D/A-I. The selection of an appropriate host solvent, the use of solvent mixtures, screening of different ratios and concentrations of the blend components, thermal annealing, and solvent-vapor treatment [99] are among the most reported approaches that have been developed to gain control over the active layer morphology.

Another widely investigated possibility consists in the dispersion of a third component into the binary D/A blend system. Third-component materials can be polymers or small molecules, liquids or solids, semiconductors or insulators, and their incorporation into OSCs can result in a series of kinetic and thermodynamic effects during and after the film formation. From a kinetic point of view, adding a third component into a BHJ blend leads to a decrease in diffusion coefficients of the D/A constituents, and to a delay of both nucleation and growth rates [100,101]. Thermodynamically, the increase in entropy upon mixing favors a homogeneous mixture over phase separation and facilitates the formation (and preservation) of a fine-blend nanostructure. Beyond these effects, the third component can also play more specific roles within the blend, such as that of a second donor or acceptor, a co-solvent, an energy cascade linker, [102] a nucleating agent, and so on. In this review, we categorize and report the role of the third component as a “Compatibilizer” (CB).

In polymer chemistry, the term “compatibilization” can be defined as a process by which interfacial properties of an immiscible blend are enhanced, while reducing the interfacial tension, stabilizing morphology, and increasing adhesion between the two phases [103]. Historically, the most popular strategy of compatibilization has been the addition of a premade compatibilizer with a good structural affinity toward the blend components. In the context of OPVs, such a compatibilizer is, in most cases, a copolymer featuring a blocky structure, with one block miscible with one component of the blend, and a second block miscible with the other, thereby facilitating their interaction and aiding adequate dispersion [104].

**Figure 2:**
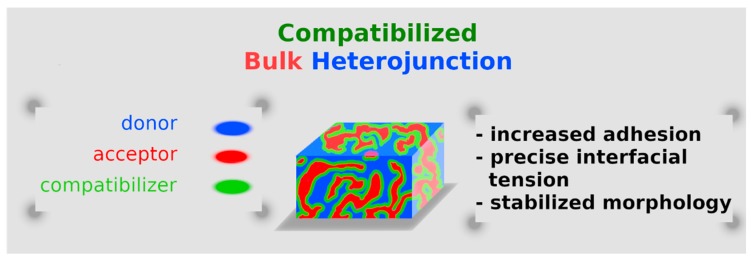
Pictorial description of a compatibilized Bulk Heterojunction (BHJ). The role of the compatibilizers (CBs) in the field of organic photovoltaics broadly matches the definition of compatibilizer in polymer chemistry.

However, since the key requirement is interfacial activity, the CB concept is not limited to copolymers with identical chain segments as those of the blend components, but can be extended to all those chemical species capable of interacting with the blend constituents through, for example, hydrogen bonding, dipole-dipole interactions, dipole-ionic, Lewis acid-base, etc. As a general criterium, they should be inert toward the components of the blend; this inertia should be necessarily seen as chemical inertia. Then, they should be selected/designed in such a way as to maximize miscibility with a specific component of the blend. Even if not strictly necessary, they should preferentially be located at the blend interface, in order to anchor the neighboring phases and maximize the compatibilizing effect. Moreover, their boiling point should be higher than that of the host solvent, so that CB is still driving the formation of D/A-I even when the solvent is partially or totally evaporated, and their concentration in the blend should be minimized in order to prevent undesirable side effects (e.g., micelle formation and higher viscosity). Last but not least, compatibilizers should helpfully provide stability to the blend they are dissolved in (Figure 2): OPVs’ stability is a crucial element for their success in the market [89], and several long-term degradation phenomena could undermine the efficiency of such organic electronics (absorber layer bleaching, thermal stress, degradation in the dark, etc.) [59]. Although innovative materials could certainly help in overcoming these issues [105], compatibilizers become extremely valuable, as they permit the reuse of already established chemistry and materials.

Structure-wise, most of the donor and acceptor materials employed in OPVs are aromatic systems; among the most studied D/A couples, the combination of poly(3-hexylthiophene) (P3HT), and [6,6]-phenyl-C_61_-butyric acid methyl ester (PC_61_BM) is representative of a considerable portion of the literature [88,106,107]. As a logic consequence, CBs usually possess aromatic moieties, but their π-network is typically limited in width in such a way as to avoid them from absorbing visible light and from possibly competing with the photoactive layer in light harvesting. As it will be further discussed in the next section, typical molecular skeletons resemble fullerenes [108,109] and naphthalenes [110]. Nevertheless, some examples of totally different molecular structures (also with non-conjugated skeletons) that can also act as effective CBs in BHJ-OSCs are provided, as well, in support of a broader definition of compatibilizer.

CBs are widely described in the literature, although often with ambiguous terminology. A deep analysis of scientific reports reveals that “additives”, “modulators”, and “nanostructuring agents” are used, as well as, more rarely, “dopants”; in some borderline cases, ternary OPVs simply correspond to binary D/A systems with a CB. The aim of this review is to provide the reader with a satisfying overview of the most recent and representative approaches targeting at the generation of optimal D/A-I by means of a minority molecular/polymeric component, able to create an interface compatible with both the given donor and acceptor materials, hence to act as an effective compatibilizer.

The available literature offers up-to-date evidences that the search for new CBs has not stopped yet; this is due to a certain degree of empiricism, which rules the investigation of D/A blends. However, recent papers point out how Hansen solubility parameters [111] can be used for guiding the selection of the appropriate CB molecule [112,113,114]. Scientists continue to study with similar enthusiasm both polymeric CBs and small molecules. Advantages and disadvantages will be pointed out when discussing specific examples, but the choice is mainly driven by processability issues and the specific D/A materials, in the end. It has to be underlined how CBs can be used for tuning D/A-I by playing around two key points: electronic levels position and morphology. In general, a specific CB follows one of these two potential approaches; nevertheless, the reader should be immediately informed that the second approach has been more widely scanned and reported.

Although OPVs have been greatly studied since the 1990s, compatibilizers chemistry became an independent and clearly defined research field only in the last two decades; it was in 2006 that a pioneering study by Sivula et al. demonstrated the potential of block copolymers as morphology modulators in OPV applications [115]. As the definition of compatibilizer is still quite blurry in several scientific reports, if not completely ignored, it is important to draw the attention of the scientific community over this fertile topic. The declared purposes of the present review are several: (i) to underline and advertise the importance of a single unambiguous definition of CBs, thus helping researchers to focus on this research field and prevent the future literature from becoming fragmentary; (ii) to offer a broad survey over the last decade reports, in order to reveal the growth in compatibilizers studies and underline the current trends, as well as some investigations which rapidly lost interest; and (iii) to update what already under the lens in some previous reviews, which are incomplete just due to the rapid shift forward of scientific research. Related to the last point, some reviews are warmly recommended as sources of historical information for the thoroughness of the specific topic therein discussed [116,117,118,119]; these last two reviews were an important source of information [120,121], and a further stimulus to bring a clear and well-defined definition of compatibilizers to the general audience and provide a useful guideline in the investigation of this fertile field of research. The central part of the review is Section 2, where compatibilizers are discussed and classified accordingly to their polymeric or molecular nature. These two principal subsections are conveniently further divided into smaller paragraphs, in order to separately collect and discuss materials possessing common fragments in their structures. In most of the cases, the discussion of a specific CB material is accompanied by the description of OPVs based on the employ of such compatibilizer; the typical parameters used for the characterization of OPVs (open circuit voltage, V_oc_, short-circuit current density, J_sc_, fill factor, FF, and power conversion efficiency, PCE) are reported, when possible, in order to provide an idea of how the compatibilizer (positively) affects the behavior of the device.

## 2. The Compatibilizers

CBs’ universe comprises a variety of functional materials; generally speaking, CBs’ library is continuously enriched by new materials due to a combination of events. On one hand, the design/selection of new CBs is mainly driven by empirical choices; a part of the literature is devoted to a never-ending trial-and-error process, where new candidate materials are obtained by little changes in the molecular skeleton of a well-established compatibilizer, looking for better performances [122]. On the other hand, the molecular skeleton and properties of a compatibilizer should preferably resemble those of either the D- or the A-material; since research continuously produces new candidate D/A-materials to be integrated in OPVs, CBs research follows a parallel evolution.

**Figure 3:**
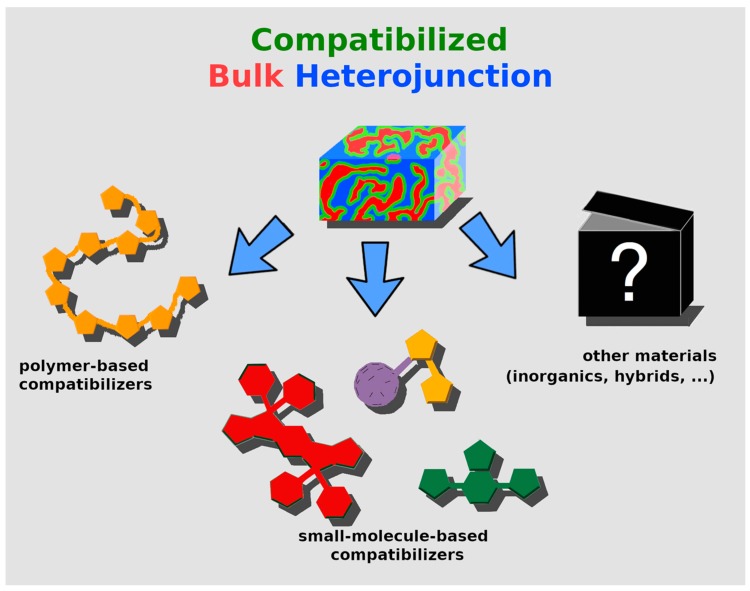
Different classes of CB-materials tested for improving the performance of BHJ-based devices.

In the following sections, a line is traced between two different universes, aiming at ordering the vast number of reports appeared in the literature, helping the reader in understanding the actual trends in research, and simplifying the choice of a specific CB-material for his/her own research purposes. In principle, we can slit the literature in two macro-areas, high-molecular-weight CBs (i.e., polymers) and low-molecular-weight CBs (i.e., molecules), which will be detailed in specific sections. There are some examples in the literature concerning other classes of materials (hybrid species and inorganic compounds [123,124], as summarized in Figure 3), but they are not discussed in detail, since our attention is directed at organic materials. In order to show the gradual progress made by the global research community, the discussion follows the chronological appearance of certain materials in the literature.

### 2.1. Polymers

Polymer chemistry is a fundamental part of OPVs background [125,126]. Some of the most employed materials as donor or acceptor partner possess polymeric structures, such as P3HT [127], poly(*p*-phenylene vinylene) [128], or their derivatives, like block copolymer P3HT-*b*-PFTBTT [129]. Polymers offer interesting advantages from a practical point of view, such as easier processability, even though it is not possible to perform a precise fine-tuning of their properties (contrary to what can be done with discrete molecular scaffolds through chemical modifications). Polymers have been widely tested, not only as active layer materials, but also as CBs, and the intrinsic properties of polymeric CBs evolved in a similar way compared to those of photoactive materials, at least from a structural point of view.

Structural moieties are a key point of the discussion, since we can operate a distinction among the examples reported in the literature, considering the common features reported: Polymers based on thiophene-like repeating units, polymers containing thiophenes and fullerenic fragments (both C_60_ and C_70_ derivatives) and polymers based on precursors which do not belong to any previously mentioned class.

**Figure 4:**
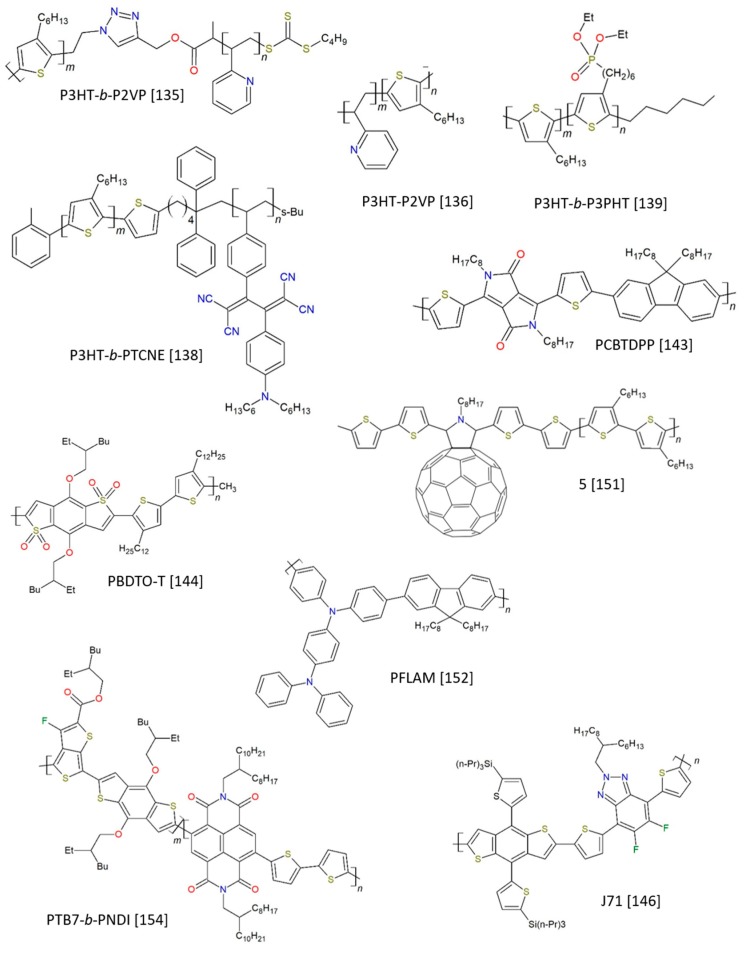
A pictorial collection of the most representative polymeric CBs described in the subsequent sections. CBs are named according to the label provided by the authors in the cited article.

Emphasizing the presence of thiophenes and fullerenes is important to underline the mechanism operated by CBs, that is to facilitate the formation of a compatibilized interface between the donor and acceptor phases through major interaction with one specific blend component. Since the OPV literature is populated by a plethora of thiophene- and fullerene-based materials, it is not surprising to find them as major pillars of the CBs universe. Here, it is worth underlining that the combination of P3HT and PC_61_BM is one of the most tested D/A combinations [130]. Before reviewing the literature focusing on CB-polymers, we would like to mention that Figure 4 presents a collection of some of the most representative structures among CB-polymers, and Table 1 has been provided to the reader in order to summarize the most relevant information and offer the chance of getting a quick overview on the following Section.

#### 2.1.1. Thiophene-Containing Polymeric CBs

D/A-I can be a complex intertwined interface to be engineered, especially in the case of BHJs, where materials are casted at the same time as a blend. An interesting solution to the problem comes with the use of rod-coil block copolymer, possessing features which aim at its co-crystallization with one of the blended components; this is the case reported by Han et al. [131], who specifically designed a block copolymer (P3HT-*b*-PTMSM), featuring a P3HT block, in order to favor the co-crystallization process with the homopolymer P3HT chains, and a second portion of poly[(3-trimethoxysilyl)propyl methacrylate] (PTMSM) as the minority block. This second component, sitting at the edge of crystalline P3HT microdomains, further evolved after thermal treatment, inducing the formation of a passivating layer at the interfacial regions between P3HT and PCBM. Silyl groups are lately converted into SiO_x_-like residues, which efficiently limit hole diffusion into the acceptor layer and thereby depress the recombination of charge carriers, affording a PCE enhancement of about 50% over the devices without the CB-polymer. It is opportune to highlight here that the authors do not ascribe the observed improvements to a compatibilizing effect in the strict sense of the term used in polymer chemistry (meaning in terms of enhanced miscibility and more favorable phase separation); indeed, the blends with and without the block copolymer exhibit almost the same temperature and enthalpy of melting, and similar phase-separation behaviors. However, in this review we would like to provide a broader definition of compatibilizer, which includes the block copolymer reported by Han et al. and, in general, all those polymers and molecules whose interfacial activities can somehow stabilize and/or enhance the performance of BHJ blends by affecting the morphology and/or the electronic and charge transfer processes at the interface.

Another strategy to control the D/A-I consists in functionalizing the P3HT-backbone of the polymeric CB and engineering the fragment destined to interact with the acceptor partner phase. AB-alternating side-chain-functionalized poly(thiophene) additives have been tested, again, in combination with a P3HT:PCBM blend [132]; although different pendants containing aromatic fragments are introduced, the incorporation of up to 10 wt.% CBs does not interfere with the advantageous formation of P3HT lamellae. Collected data justify the increase in PCE even at low CB addition (0.25 wt.%) by evoking the introduction of a dipole at the polymer/fullerene interface operated by the side-chain aromatic moieties; this dipole depresses the charge carrier recombination rate. Some trends are disclosed, with the perfluorophenoxy-containing polymer as the most efficient CB, yielding a 28% increase in PCE when incorporated into the P3HT:PCBM BHJ at 0.25 wt.%. Another example of modified P3HT, Chen and coworkers introduced hydroxyl groups at the end of polythiophene chains, revealing an improved control over P3HT:PC_61_BM interface morphology [133]. A combination of SEM and AFM images confirms the reduced dimension of fullerene domains, while electrical characterization evidences superior PCE values.

**Figure 5:**
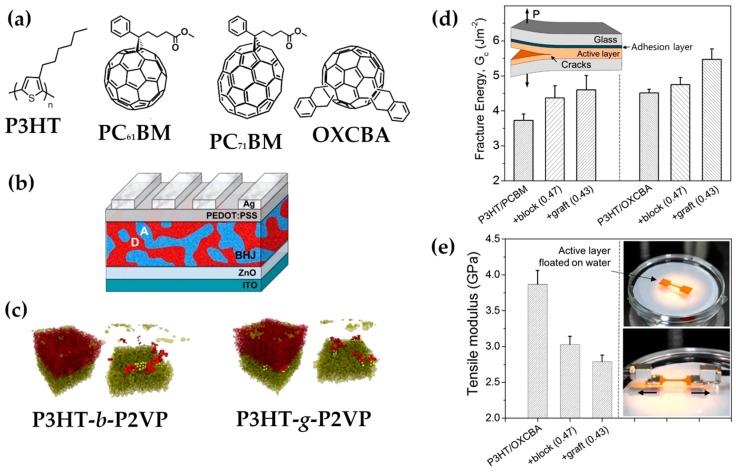
Poly(vinylpyridine)-based CBs. (**a**) Chemical structures of the polymer electron donor (P3HT) and fullerene-derivative electron acceptors (OXCBA, PCBM, and PC71BM). (**b**) Scheme of the inverted solar cell. (**c**) Models for the molecular configurations at the D/A interfaces for the poly(3-hexylthiophene)-block-poly(2-vinylpyridine) (P3HT-*b*-P2VP) and the poly(3-hexylthiophene)-graft-poly(2-vinylpyridine) (P3HT-*g*-P2VP). Effect of the P3HT-*b*-P2VP and P3HT-*g*-P2VP on (**d**) fracture energy and (**e**) tensile modulus of P3HT:PCBM and P3HT:OXCBA BHJ films. Reprinted with permission from Reference [135]. Copyright 2014 American Chemical Society.

Keeping in mind a similar purpose, but with a different structure and mechanism behind the improvement, Renaud and collaborators develop a P3HT-*b*-P4VP block copolymer [134]. A comparison between OPVs prepared with and without the compatibilizer reveals that, after thermal annealing, and under the same experimental conditions, PCE value increases by an impressive 60%, up to a maximum of 4.3% at a CB concentration of 8 wt.%. The explanation of the results is based on Grazing Incidence X-ray Diffraction (GIXD) measurements, showing that, even if the P3HT phase crystallinity is slightly decreased, the crystallites now prefer to assume a faces-on orientation, thereby benefiting the process of hole extraction toward the corresponding electrode. Moreover, PCBM crystallization is efficiently inhibited, resulting in a size decrease of PCBM microphases and an increase in the interfacial area available for exciton dissociation. A similar mechanism can be attributed to the block copolymer P3HT-*b*-P2VP, where a P3HT portion is combined with poly(2-vinylpyridine). The P3HT portion is again employed to modulate the interfacial interactions between the conducting polymer and a second partner. Pyridine residues are able to establish positive interactions with a variety of partner phases, ranging from fullerene acceptors (including bis-adduct fullerenes) [135] to inorganic materials, such as TiO_2_ nanorods [136]. In the first case, since poly(vinylpyridine) is known to establish strong supramolecular interactions with electron-deficient fullerene molecules, this feature is usefully exploited to reduce the interfacial tension, enhance the resistance against the crack growth, and prevent the debonding between the two phases (Figure 5). In the latter case, the CB-polymer plays a tangible role in the efficiency of the charge-separation process and produces a great photoluminescence quenching. Similar considerations can be extended to the work by Chen et al. [137]; however, the authors do not evoke H-bonding networks to justify the efficient compatibilizing effect induced by the copolymers, but rather they propose that the pyridine units at the D/A-I facilitate the exciton dissociation, finally resulting in an increase of PCE.

Some reports start to point attention to diblock copolymers, which are not a “merely” interfacial compatibilizer, but could contribute to the alteration of the blend energy levels. For example, Fujita et al. realized P3HT:PCBM OPVs where two different CBs were tested: the AB diblock copolymer, P3HT-*b*-PTCNE, and an ABA triblock copolymer, PTCNE-*b*-P3HT-*b*-PTCNE [138]. Both are based on the combination of a P3HT portion and polystyrene derivative with D/A units (PTCNE). Between the lines of their report, it is evident how P3HT crystallinity is enhanced, similarly to what previously described, and an increase in the photovoltaic parameters, such as the J_sc_, the FF, and consequently the PCE, are reported. Moreover, spectrophotometric measurements suggest that the presence of CB-polymer introduces suitable energy levels for the smooth energy transfer via block copolymers in the P3HT:PCBM blend films. Notably, superior compatibilizing performance is confirmed for the ABA triblock copolymer (PCE increased by 6.6%) when compared to the AB diblock material, due to a change in the crystalline domain orientation from “edge-on” to “isotropic” in the area where PCBM domains are more separated.

**Figure 6:**
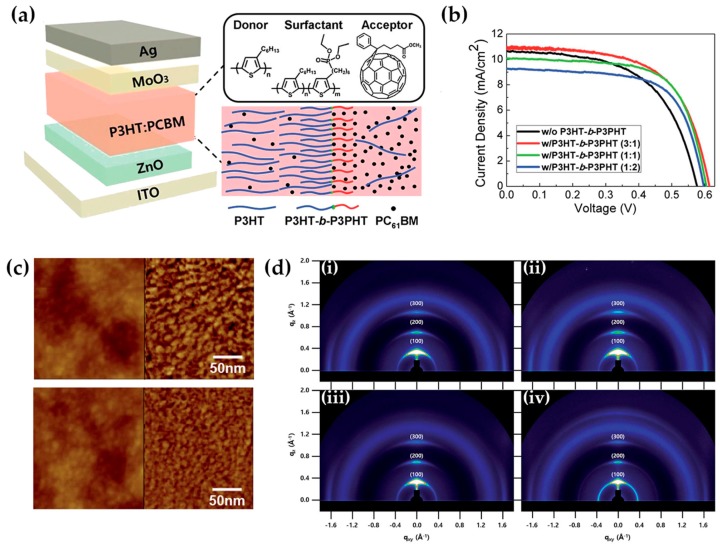
Amphiphilic block copolymer-based CBs. (**a**) Addition of P3HT-*b*-P3PHT block copolymers into the P3HT:PC_61_BM film and diffusion at the P3HT:PC_61_BM interface. (**b**) Comparison of the J-V curves of the P3HT:PC_61_BM control device and the P3HT:PC_61_BM device blended with 5 wt.% P3HT-*b*-P3PHT diblock copolymers. (**c**) AFM topography (left) and phase images (right) of the (i) P3HT:PC_61_BM; (ii) P3HT:PC_61_BM blended with 5 wt.% P3HT-*b*-P3PHT (3:1). (**d**) GIWAXS characterization of (i) P3HT:PC_61_BM, and P3HT:PCBM blended with (ii) P3HT-*b*-P3PHT BCP (3:1), (iii) P3HT-*b*-P3PHT (1:1), and (iv) P3HT-*b*-P3PHT (1:2) thin films. Reproduced from Reference [139], with permission from the Royal Society of Chemistry.

Improvements in these studies are brought by another block copolymer, P3HT-*b*-P3PHT [139], which is able to diffuse at the P3HT:PCBM interface and enhance the miscibility between the two blend constituents, inducing an increase in the interfacial area between the P3HT phase and the smaller but more abundant PCBM isles (Figure 6). Here, the pivotal role has to be attributed to the introduction of the phosphonate group in the hexyl side chains, which provide an amphiphilic nature to the block copolymer, lower melting temperature, and a reduction in the rod-rod interactions, aiming at better dispersion into the blend matrix.

Block copolymers incorporating both P3HT and poly(styrene) portions are well-known polymeric CBs, widely reported to improve the morphology of P3HT blends in combination with PCBM [140] or other fulleropyrrolidine derivatives [141]. An “exotic” approach has been recently referred to by Mohammadi-Arbati and colleagues, who have reported the combined addition of a rod-coil block copolymer comprising P3HT and polystyrene (P3HT-*b*-PS), and reduced graphene oxide nanosheets grafted with regioregular poly(3-hexylthiophene) (rGO-*g*-P3HT) as compatibilizers in a typical P3HT:PCBM blend [142]. A combination of data acquired by XPS, TEM, and AFM supports the hypothesis that the CB localizes at the interface, with P3HT portions from the copolymer interacting with the P3HT residues grafted onto rGO-*g*-P3HT. The so-made interface generates new pathways for the charge carriers to rapidly flow through, ending up in a dramatic increase of their charge mobility values. The final device further benefits from thermal annealing at 120 °C, which produces hole and electron mobilities as high as 9.8 × 10^−4^ and 2.7 × 10^−3^ cm^2^, respectively. The beneficial impact of the new architecture is confirmed by the high values of J_sc_, FF, open-circuit voltage (V_oc_), and PCE parameters, which are 12.98 mA/cm^2^, 0.69 V, 68%, and 6.09%, respectively.

One very recent example concerning a P3HT:PCBM OPV device looks interesting for opening discussion over some “confusing” terminology. In the paper from Xu and coworkers [143], they describe an OPV based on the classic architecture P3HT:PCBM, where a compatible low-bandgap polymer is added, PCBTDPP, in order to act as a “bridge” between the main donor and acceptor units, for an increased flow of charge carriers. Interestingly, using comparable preparation conditions for a device, including the compatibilizer, and a second one acting as benchmark with no addition of PCBTDPP, the PCE is measured as high as 5.28% versus 4.67% for the benchmark device. The authors attribute the improvement in performance to the high mobility and extended absorption by the polymeric CB, plus improved exciton migration/dissociation and energy transfer. By the way, the solar cell is here defined as “ternary”; in our opinion, since they document that the optimal CB-concentration was as low as 0.2 wt.%, it would be worth underlining how the terminology can be somehow confusing and that the community should, for instance, operate some criticism in order to establish more well-defined phrasings.

The usefulness of polymer-CBs is nicely explored also in the case of an immiscible binary blend based on the combination of P3HT and polymeric derivatives of benzothiadiazole, PCDTBTs; here, in [104], a block copolymer based on both the P3HT and PCDTBT scaffolds is developed on purpose. Optical micrographs immediately reveal that macrophase separation occurs for the photoactive blend in absence of any compatibilizer, with P3HT domains larger than 50 μm; upon progressive addition of the compatibilizer, the presence of only 1% SFBCP-13 reduces P3HT domain size by the half (ca. 30 μm), whereas further increase brings the domains down to ∼3 μm (20 wt.%). Further additions do not produce any defined phases network. GIXS measurements show that the presence of CB-polymer induces a beneficial reorientation of P3HT crystallites, further improving the morphological profile of the blend. Consequently, increased photoluminescence quenching and enhanced solar cell performance are observed.

**Figure 7:**
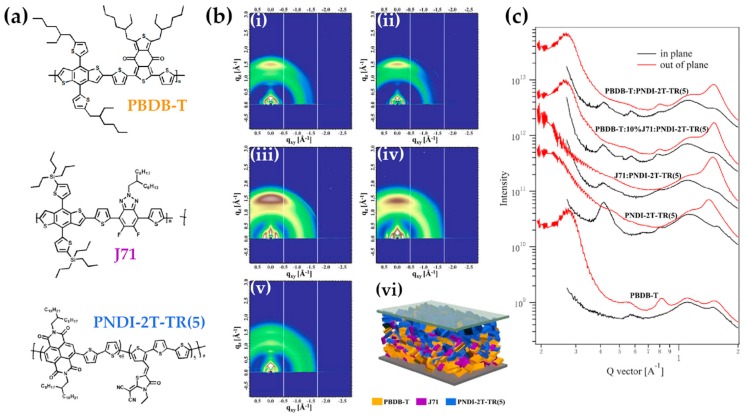
J71 as CB in all-polymer solar cells**.** (**a**) Chemical structures of PBDB-T (donor), J71 (CB), and PNDI-2TTR (acceptor). (**b**) GIWAXS characterization of (i) PBDB-T:PNDI-2T-TR(5), (ii) PBDB-T:10% J71:PNDI-2T-TR(5), (iii) J71:PNDI-2TTR(5), (iv) PNDI-2T-TR(5), and (v) PBDB-T; (vi) pictorial representation of J71 localization into the PBDB-T:PNDI-2TTR film. (**c**) In-plane and out-of-plane line extracts from the GIWAXS patterns reported in (**b**). Reproduced with permission from Reference [146]. Copyright 2019 American Chemical Society.

In the work from Lei and colleagues, benzo[1,2-b:4,5-b′]dithiophene1,1,5,5-tetraoxide (BDTO) is originally used as a starting core for the production of two polymer materials, namely PBDTO and PBDTO-T, where the latter is the oxidized (and electron-poorer) modification of the first one [144]. When employed as compatibilizers for the PBDB-T:IT-M blend, the smoothing role played by CBs at the interface, combined with a doping effect and high charge mobility, allows for an increase in the PCE value from 10.31%, yielded by the benchmark PBDB-T:IT-M device, to 11.12% and 11.47%, when using PBDTO and PBDTO-T, respectively. Benzothiophenes are a recurrent core for other compatibilizers, such as PBDB-T [145]; energy levels derived by optical spectroscopy suggest its employ as compatibilizer for dimalononitrile polymer DRCN5T and fullerene acceptor PCBM. The CB improves DRCN5T miscibility and enhances the crystallinity, acting as a good morphology and performance modulator. With the highest doping addition of 20%, PCE rises from 7.74% to 9.45%, J_sc_ from 14.06 to 15.98 mA/cm^2^, and FF from 58.56% to 65.72%. Ultimately, 20% is a sensibly high ratio, which is the reason why here the definition of PBDB-T as compatibilizer is very weak, and probably it would be more honest to define those devices as examples of ternary OSCs. Better performances are offered by a different member of the family of benzothiophenes-polymers, namely J71. In the example [146], 10 wt.% addition of J71 to the PBDB-T:PNDI-2T-TR(5) blend leads to V_oc_ = 0.88 V, J_sc_ = 14.63 mA/cm^2^, FF = 71.05% and PCE = 9.05%. The study evidences, once more, the morphological implication of such CB in the modulation of the mixing interface and the facilitation of electron transfer phenomena. In order to prove these speculations, it is important to consider the change in the glass transition temperature (T_g_); using the Fox equation, the authors initially hypothesize the desired miscibility between PBDB-T and J71, which is lately confirmed by dynamic mechanical analysis (DMA) data and differential scanning calorimetry (DSC); a combination of TEM and AFM images confirms it, as well the reduced dimension of the phase domain size (Figure 7).

#### 2.1.2. Thiophene-and Fullerene-Containing Polymeric CBs

As discussed in the previous section, several compatibilizing polymers include thiophene rings in their backbone, or even entire P3HT portions, as long as polythiophenes are used as the donor materials (or, less frequently, the acceptor component), in order to maximize the interactions and favor morphological control over mixing interfaces. Consequently, a logical choice for further evolution of compatibilizers consists in the integration of both thiophenes and fullerene fragments onto the same polymer scaffold. There are some recent and interesting examples, although the major difficulties in the synthesis and the limited increase in the performance of the corresponding devices diverted more attention toward simpler polymers, if not on discrete molecules.

Some first reports are dated back to the beginning of the decade. Rattanathamwati and collaborators envisaged the possibility of preparing block copolymers based on the combination of P3HT, polystyrene, and fullerene-grafted polystyrene [147]. Unfortunately, the results obtained for P3HT:C_60_ were obscured by very low PCE values, although some improvement was revealed for samples prepared with the addition of CB-polymer. The attempts reported by Lee et al. are definitely more successful. In their work [148], the blend under investigation is a typical P3HT:PCBM mixture, with the compatibilizing diblock copolymer P3HT-*b*-C_60_ simply designed around the employ of regioregular P3HT and commercial PCBM. As evidenced by optical microscopy, the addition of 2.5 wt.% CB-polymer helps in inhibiting macrophase separation and creating a more homogeneous layer, with the diblock polymer localized at the interface. The resulting PCE increase is quite limited, with the best value (obtained after annealing the sample for 15′ @ 150 °C) equal to 3.19% vs 3.05% for the CB-free sample.

Biciocchi and coworkers develop a block copolymer based on the combination of a polyfluorene fragment and fullerene portions (F6T2-*b*-PS(C_60_) and F6T2-*b*-PS(PCBM)) as compatibilizers in bulk heterojunction solar cells [149]. In the example, low performances are shown by the tested devices. However, it is worth mentioning that the authors plan a long cycle of tests for their devices, up to four months, and underline how, although the presence of compatibilizing polymers did not boost up the efficiency of the devices, they definitely contributed to the stability of the OPV, producing stable performances within this long time window, if compared with benchmark solar cells.

Some materials are also documented by Kakogianni et al., based on a P3HT:PC_71_BM scaffold [150]; moreover, they made a complete characterization of the materials, showing great potential for OPV technology application, as no other tests on a real device are reported up to date.

**Figure 8:**
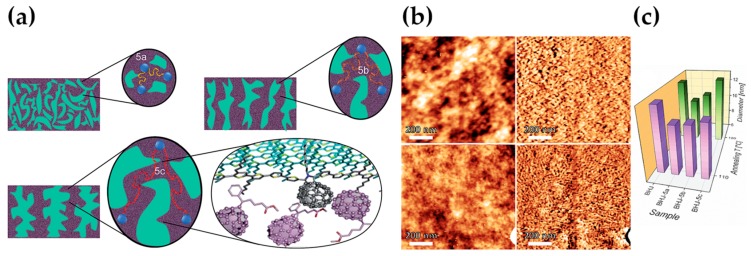
CBs based on fulleropyrrolidine copolymers bearing oligothiophene substituents at different length. (**a**) Domain size distribution regulated by the copolymers 5a–c. (**b**) The AFM characterization (morphology and phase) of the deposited films without (at the top) and in presence (at the bottom) of the 5b copolymer (2 wt.%). (**c**) Investigation on the P3HT domain size for BHJ, without copolymer and in presence of different copolymers (5a, 5b, and 5c). Thin-film characterization (**b**,**c**) was carried out after annealing at 110 °C for 5 min. Reproduced from Reference [151], with permission from the Royal Society of Chemistry.

Our group has been devoted to the study of OPVs for a long time; in a recent report [151], a new library of copolymers resulting from a combination of oligothiophene chains and fullerene pendants is synthesized accordingly to easy and inexpensive one-step synthetic approach. The copolymers are incorporated into typical P3HT:PC_61_BM BHJ and good control over phase-separation process is achieved, as shown by fluorescence measurements, X-ray diffraction data, and AFM images analysis, without further affecting the BHJ optoelectronic properties, and by using a relatively low concentration of CB (2 wt.%) (Figure 8). Besides the importance of thermal annealing as a tool for the modulation of domains size, it is quite interesting to point out the high control over the difference in the oligothiophene chains length among the tested compatibilizers; by employing copolymers containing oligothiophenic chains with a size of about 8 nm, the power conversion efficiency (4.46%) and short current density (J_sc_, 16.15 mA/cm^2^) resulted to be the highest so-far-reported values for P3HT:PCBM solar cells processed on plastic substrates.

#### 2.1.3. Polymeric CBs without Thiophene and/or Fullerene-Fragments

The search for new compatibilizers brought the exploration of materials with a high degree of novelty that do not necessarily resemble the D/A components employed for the realization of OPVs. This is the case for PFLAM, an electron-donor polymer based on the repetition of fluorene and triphenylamine units [152]. This material, characterized by elevated hole mobility (10^−3^ × cm^2^V^-1^s^-1^), is tested as CB for a common P3HT:PCBM blend; the addition of PFLAM results in higher hole mobility, with an elevated efficiency gain for such a device, quantified in a remarkable 34%. The power conversion efficiency of this device reaches 3.3%, using 3 wt.% CB; a further increase in CB concentration lowers the PCE due to the excessive hole transport, disturbing the charge balance in the device. Similarly, Chi and collaborators propose THC8, a bifunctional polymer able to improve charge transport and possibly broaden the spectral absorption [153]. TCH8 structure is characterized by a combination of di-triarylamine and fluorene moieties, high absorbance in the visible range (λ_max_ = 420 nm), and strong fluorescence centered at 510 nm. Morphological aspects are important, as well, and the mixing interface modulation operated by this CB provides improved charge transport properties for P3HT:PCBM-based devices. Final tests with optimal CB addition of THC8 (9 wt.%) return more performing devices compared to the benchmark OPV with no additive, with improved J_sc_ (from 10.55 to 11.97 mA/cm^2^), FF (from 48.31% to 52.54%), and PCE (from 3.10% to 3.88%).

Another innovative block copolymer is reported by the team of Rafael Verduzco, who investigate a combination of poly(thieno[3,4-b]-thiophene-*co*-benzodithiophene) and poly(naphthalene diimide), namely PTB7-*b*-PNDI [154]. In their study, a low amount (3 wt.%) addition of the all-conjugated block copolymer CB in PTB7:PC_61_BM blends results in an increased device performance, with V_oc_ = 0.82 V, PCE = 5.1% and FF = 52.5%. Using a pure PNDI CB-polymer, the authors can discern the contribution of the naphthalenic material, which exhibits a beneficial impact on device PCE, predominantly through an increase in the short circuit current and fill factor.

Some last words should be spent for the simple and commercial poly(2-vinylpyridine) (P2VP); Lee and colleagues employ this polymer as an additive for a series of OSCs based on benzodithiophene donors and a variety of acceptors (PCBM or naphthalene diimide-based polymer) [155]. This report is mentioned at the end of the section, since, this time, the interface between the BHJ and the cathode/metal oxide (MO) layers was investigated. All the tested cells show improved conversion efficiency and enhanced stability under ambient conditions. These findings are justified by the authors as follows: The vertically segregated P2VP layer on the MO surface acts as an effective passivating layer, preventing oxygen and water chemisorption, suppressing the related interfacial traps, and consequently leading to reliable and constant electric characteristics.

### 2.2. Molecules

Discrete molecules are often reported as useful compatibilizers for OPVs. The advantage of employing small molecules instead of polymer chains lies in their discrete nature: They can be easily modulated in terms of photophysical behavior by means of well-known and established chemical procedures, so that libraries of compounds can be rapidly prepared and tested. Similar to what was previously described in the field of polymeric compatibilizers, structural design comes from a combination of processing needs and empirical resemblance by CB-molecule to the D/A structures.

**Figure 9:**
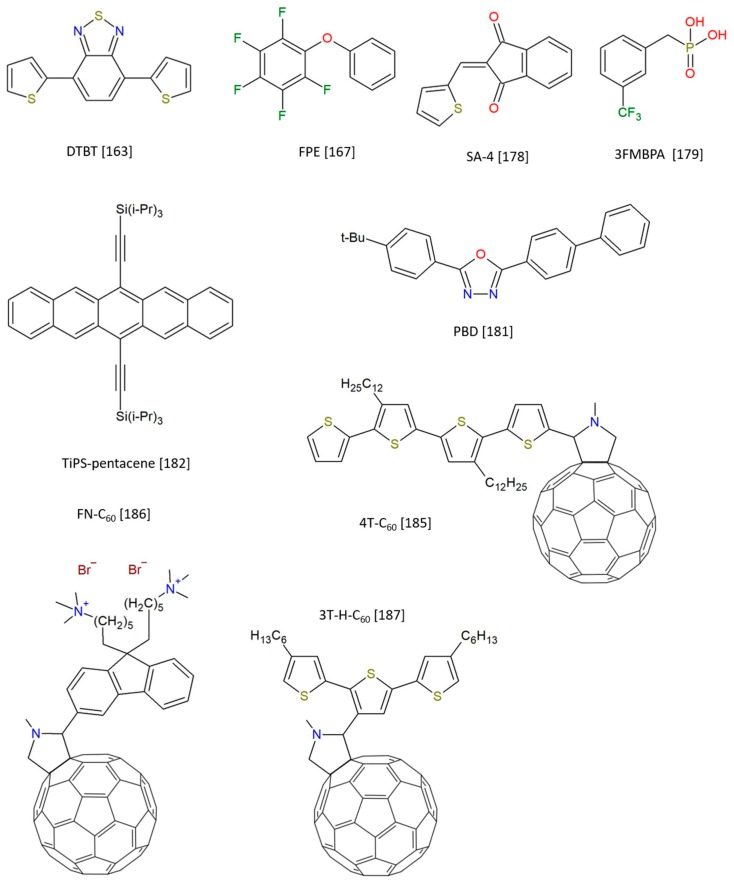
A pictorial collection of the most representative small-molecule-based CBs described in the subsequent sections. CBs are named according to the label provided by the authors in the cited article.

By the way, this assumption is not always verified, and this is the reason why it is worth rationalizing the following discussion by introducing some guiding criteria. According to the number of available reports up to date, molecule-based CBs can be classified according to their following characteristics: aromatic core, fulleroid structure, and non-conjugated skeleton. As already done in the previous Section focusing on CB-polymers, a figure showing some of most representative CB-molecules is provided (Figure 9), together with a table summarizing the most relevant information (Table 2).

#### 2.2.1. Aromatic-Core-Based Compatibilizers

It is worthy to start this section by introducing some high-molecular-weight and branched molecules, which can serve as “bridging” examples between polymer universe and molecules libraries. In a recent report, Chen et al. describe a novel triazole-cored, star-shaped, conjugated molecule TDGTPA, to be used as compatibilizer in an inverted P3HT:PC_71_BM solar cell [156]. From a structural point of view, TDGTPA comprises a central triazole core, 2,5-thienyl diketopyrrolo-pyrrole units as π-conjugated bridges and responsible for the interaction with the D/A materials, and peripheral *tert*-butyl-substituted triphenylamine acting as donor unit. Interactions drive TDGTPA to influence the interface with the underlying ZnO layer instead of that between P3HT and PC_71_BM. The beneficial action of the CB-molecule may be foreseen in an improved physical contact at the metal oxide/photoactive layer interface and partially suppressed charge trapping/recombination mechanism, which are generally depressing the device performance. The improved efficiency of the compatibilized devices (3.38%) vs. the one with no CB (2.92% and 3.38%) is then attributed to the establishment of local interfacial dipoles operated by ethylene oxide pendants, which are actively modifying the electrode work function, thus facilitating electron extraction processes and their injection in the outer circuit. Choosing structurally similar compatibilizers is a rewarding approach also for Jiang et al., who modulate the crystallinity of their polymeric photoactive material, resulting in an efficient energy transfer [157]. Their IT-4F contains indanedione-like fragments, as well as fused thiophene portions and a benzofluorene-like core, and closely resembles the main acceptor molecule ITIC; although the paper claims that their device should be considered as a ternary OSC, the relatively low percentage of added IT-4F (in the range of 9%), induces us to include the example in our review. The best PCE revealed for the example here reported is 13.27%, a quite high value which must be carefully considered after observing that benchmark cells showed 11.83% PCE. The action of the additive is crucial for improving the performance; nevertheless, it must be admitted that the pristine photoactive layer already shows great values.

The aforementioned examples underline that thiophenes are ubiquitously present as part of massive molecular compatibilizers, like before, but they show potential as CBs, even when the structure is extremely simple; here, Peng et al. investigate the effect of simple 3-methylthiophene (3MT) and 3-hexylthiophene (3HT) on the morphology and photovoltaic performance of polymer solar cells (PSCs) [158]. P3HT:PCBM is, once more, the benchmark D/A photoactive layer, and test devices soon reveal enhanced optical properties, with increased absorption in the 500–650 nm spectral window, and additional higher hole mobility. Morphological modulation contribution by CB-molecules can be observed by means of AFM images, with 3MT-processed blends showing phase separation at the nanoscale domain, and formation of an interpenetrating network morphology between the D- and the A-domains. Moreover, 3-HT showed great stability, providing almost identical PCE values for the device undergoing thermal annealing or not (ca. 3.30%). In parallel, 3-MT provides the best absolute performance and, remarkably, without thermal annealing step, with 3.65% PCE vs. 2.76% registered for the benchmark device with no CB. This result is particularly interesting because 3-MT can be conveniently employed, even in the case of temperature-sensitive materials for the photoactive layer.

Pyridines are well-established CBs, as well; although there are few D/A materials based on this small aromatic molecule, pyridines have an aromatic skeleton able to produce efficient π–π interactions and an advantageous basic site, which could be employed for establishing efficient hydrogen bonds. This is the concept behind the work of Xu and coworkers, who tested a couple of derivatives (i.e., 2-hydroxypyridine, 2-DHP, and 2,4-dihydroxypyridine, 2,4-DHP), with the aim to improve the nanomorphology of a classical P3HT:PC_61_BM OPV [159]. A combination of AFM and XPS data revealed a more efficient phase separation and structural organization of the polymer and fullerene domains, which was justified, considering that DHP-CBs are able to establish π–π interactions with P3HT, and drive at use N-atoms for the creation of H-bonds, together with the PCBM carboxylic residue. Although 2,4-DHP is able to create more H-bonds, the best performance is revealed for the blend containing 2-DHP, with PCE increased from 3.01% to 4.35%. Although this review points its attention over OSCs, it is worth reporting an example related to a different application field, in order to show that similar strategies can be applied in different contexts. In the following example, perovskite-based solar cells can benefit, as well, from pyridine-based additives for similar reasons. Having P3HT as hole-transporting layer, the devices show increased PCE, once again thanks to an improved microscopic order induced by 4-*tert*-butylpyridine (TBP) within the polymeric layer [160]. Pyridines also demonstrate their usefulness by improving the performance of poly(3,4-ethylenedioxy-thiophene):poly(styrene sulfonate) (PEDOT:PSS), most commonly used as anode buffer layer in bulk-heterojunction (BHJ) polymer solar cells (PSCs). In a report published by Xu and others, the overall performance of a device based on P3HT:PC_61_BM blend benefits from the addition (1 wt.%) of 2,3-dihydroxypyridine, DOH, to the PEDOT:PSS film [161]; pyridine is, indeed, able to create H-bonds with sulphonate groups from PSS matrix, thus helping to achieve a better phase separation between PEDOT and PSS, and promoting PEDOT isomerization into the more conductive quinoid form, as suggested by FT-IR and Raman spectra. Thus, the approach results in a decreased PEDOT:PSS work function (from −5.24 to −5.13 eV), higher hole mobility, and a satisfying 20% increase in the PCE compared with the PEDOT:PSS non-compatibilized device. The alkaline nature of DOH produces a desired enhancement of the thermal and air stability of the fabricated OPVs. Similar encouraging results are lately reported by the same authors [162], with DOH inducing a finer nanoscale phase segregation between polymer and fullerene domains in a PDPP3T:PC_61_BM photoactive layer, resulting in a significant PCE increase (6.36% vs. 3.76% for the reference device) after a negligible addition of the CB (0.5 wt.%).

**Figure 10:**
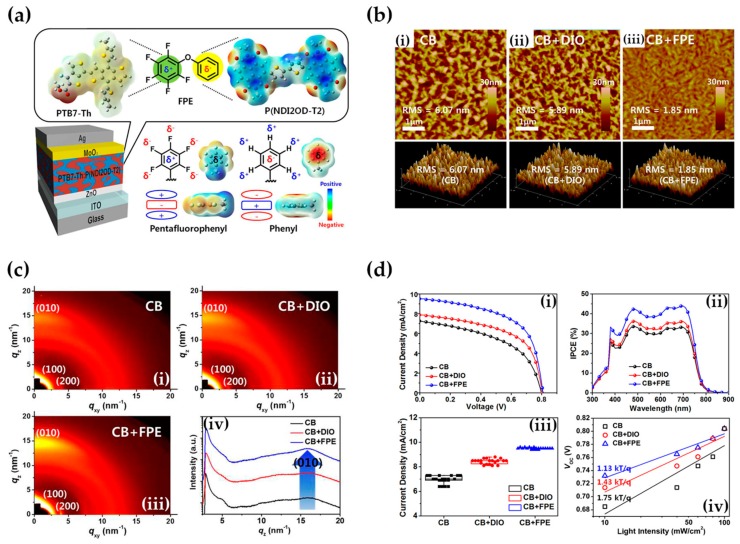
Pentafluorobenzene-based compatibilizers in all-PSCs. (**a**) Control of the interfacial morphology between PTB7-Th and P(NDI2OD-T2) blend films via electrostatic interactions with pentafluoro-6-phenoxybenzene (FPE). (**b**) AFM characterization (height at the top, 3D images at the bottom) of the TB7-Th:P(NDI2OD-T2) blend films processed with (i) CB, (ii) CB + DIO, and (iii) CB + FPE. Note that CB stays here for chlorobenzene, DIO for 1,8-diiodooctane. (**c**) GIWAXS graphs of PTB7-Th:P(NDI2OD-T2) blend films processed with (i) CB, (ii) CB + DIO, and (iii) CB + FPE; (iv) corresponding GIWAXS out-of-plane patterns for the three cases. (**d**) Photovoltaic characterization of the devices processed with CB, CB + DIO, and CB + FPE, by reporting the (i) J−V graph, (ii) IPCE spectra, (iii) statistical analysis of J_SC_, and (iv) the V_OC_ as a function of the light intensity. Reproduced with permission from Reference [167]. Copyright (2017) American Chemical Society.

Other simple and polar aromats are under the lens, and, among them, diphenyl ethers (DPEs) deserve a discussion. DPEs are funneling researchers’ interest due to their ability to promote nanostructuring within the photoactive layer and boost charge transport phenomena by suppressing recombination processes [163,164]. Unsubstituted DPE is investigated by Zheng et al. in the frame of PTB7:PC_71_BM OPVs preparation [165]; an addition of 4% *v*/*v* DPE caused a three-fold PCE enhancement (from 2% to 6%), due to an increased absorption in the visible range revealed by optical microscopy (increased absorption peak at 450 nm) formation of ordered packing of the PTB7, and reduction of PC_71_BM to the 500 nm range (as proofed by AFM). In a different study, Laventure and colleagues use DPEs for tuning the solid-state aggregation of a twisted N-annulated perylene diimide acceptor dimer [166]. AFM images support the hypothesis by revealing that photoactive layers prepared with no DPE present the molecular acceptor aggregated with fibrillar morphology, while the films processed with DPE show an evolution in the topography, from fibrillar to granular. Recently, a little step further is revealed to the community, with Kim and collaborators working on a pentafluorobenzene-based diphenyl ether (F-DPE) [167], able to govern the D/A interfacial morphology via quadrupolar electrostatic interactions between donor and acceptor polymers. AFM measurements reveal diminished surface roughness, interpenetrating morphology without large-scale phase separation and an enhanced, ordered π−π stacking with face-on orientation among the polymer chains (Figure 10).

**Figure 11:**
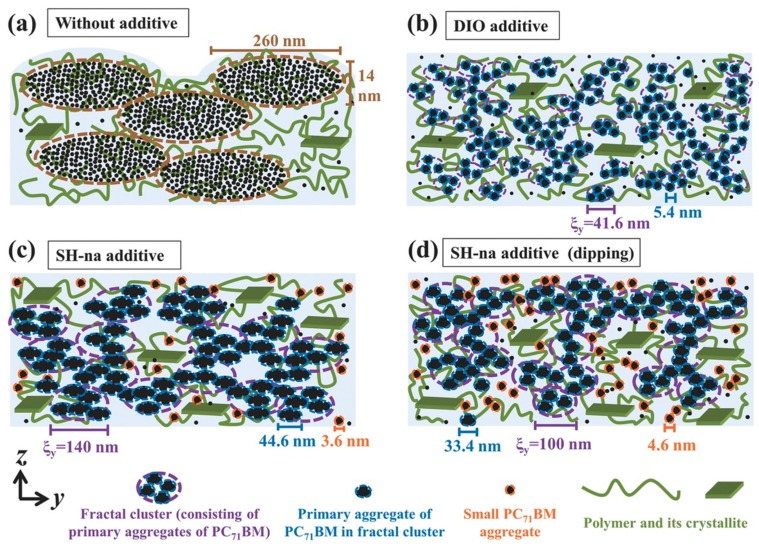
Effects of DIO (1,8-diiodooctane) and SH-na (1-naphthalenethiol) CBs on the molecular organization of PTB7:PC_71_BM active layers. (**a**) Without CBs, large aggregates of PC_71_BM are observed; (**b**) DIO suppresses large aggregation of PC_71_BM; (**c**) SH-na enhances PTB7 chain packing and multi-length PC_71_BM dispersion; (**d**) SH-na processed via dipping lead to similar results to the ones observed in (**c**). Reproduced with permission from Reference [176]. Copyright © 2016 by WILEY-VCH.

Several small benzene-derivatives have been actively tested, with little modification of the molecular scaffold by the introduction of halogen substituents, or oxygen-containing moieties [112,168,169]. Among small aromatic CBs, a benchmark molecule is still 1-chloronaphtalene (CN) [163,164,170,171,172], whose role is widely recognized as morphology modulator for PCBM-rich phases; in particular, an increase in the fraction of aggregates with “face-on” orientations compared with thin films processed without any CB has been revealed [173]. Non-halogenated naphthalenes have been proposed as a potential step forward, due to the lack of halogens in the molecular scaffold, thus paving the way for environmentally compatible processing solutions [114,174,175]. Interestingly, 1-naphthalenethiol (SH-na) has recently shown great potential in PSCs processing [176]. In the report by Jhuo et al., where the preparation of PTB7:PC_71_BM photoactive layer in presence of SH-na is discussed, the achieved PCE of 7.3% is justified by the capability of SH-na to establish hydrogen bonds with both PTB7 and PC_71_BM, resulting in the improvement of both PTB7 crystallites quality and PC_71_BM dispersion. Results are supported by a combination of GISAXS/GIWAXS data, neutron reflectivity, AFM, FT-IR, and XRD measurements (Figure 11). Moreover, final comparison with similar PSCs prepared in presence of well-known solvent additive 1,8-diiodooctane (DIO) [177] results in poorer performance (6.7%), opening the door for new simple and competitive CB-molecules. In a comparative test, dimethyl phthalate emerged as a very promising candidate for the realization of PEDOT:PSS-based OSCs, with PCE increasing by 113% in comparison to a control OSC without the processing additive [112]. Unfortunately, no other reports have been found, reporting a follow-up of their scientific investigation.

Some novelty in the field has been recently brought by Yu and coworkers, with their broad investigation focused on indanedione chemistry [178]. In their work, solid additives based on indanedione scaffolds are efficiently employed for the compatibilization of IT-4F, a molecular acceptor comprising indanedione portions, fused thiophenes, and a fluorene-like structure within the molecular skeleton. Besides the interesting values revealed for the so-made OPVs, the authors compare the PCE values obtained by adding different CB molecules. Optimal PCE values are recorded for the devices where SA-2/4 were added: Here, the molecular structures were kept as simple as possible, occasionally exchanging benzene rings with thiophenes. A depression of the performance is observed for the most electron-poor CB, featuring cyano- and fluorine-substituents directly connected to the aromatic scaffold, with the CN-substitution giving the worst results.

Small aromatic molecules are also designed not specifically for improving the D/A-I, but with the aim of enhancing the interfacial properties between electrodes and photoactive materials. Two very recent papers describe the positive effect due to the addition of relatively small and structurally simple polar aromatic compounds. In the first example, a fluorinated benzylphosphonic acid (3FMBPA) is designed as compatibilizer between the photoactive blend and the ZnO layer [179]. The article reports higher PCE in presence of the CB-molecules. In order to rationalize the results, a deep morphological characterization is performed with STEM-EDX measurements revealing that the CB is mostly localized at the ZnO:BHJ interface, while GIXRD patterns and HR-SEM images reveal high quality crystalline ZnO layer formation after its deposition. The authors suggest that the evidences are in favor of a migration of 3FMBPA, which localizes at the top of the film surface, ending in the formation of a dense passivating layer, able to reduce interfacial energy and efficiently work as trap-suppressor. Moreover, the high crystallinity of ZnO suggests that the CB acts as a nucleating agent during the atomic layer deposition process. O-defects present in the ZnO layer are suppressed by Ou et al. recurring to a benzoylthiourea [180]; in their hypothesis, formation of N-Zn bonding and the resulting reduced oxygen vacancies (as revealed by X-ray photoelectron spectroscopic, XPS) passivate O-defects. The hydrophobic nature of the CB molecule has a secondary beneficial effect, depressing the absorption of external agents such as moisture within the layer. A better charge transport ability is also achieved by means of an oxadiazole-based electron-transporting material, PBD, placed between the ZnO ETL and the photoactive layer [181]. Consequently, PCE increases from 10.8% to 11.6%, due to the simultaneous enhancement in J_sc_ and FF.

One last mention for an exotic structure with pentacene-derivatives. Wang and colleagues try to offer a novel CB-molecule, starting from electronic considerations, instead of giving priority to the morphological control of the photoactive blend. In their report [182], they introduced pentacene as the core of their CB, since pentacene is a well-known high-mobility small aromatic compound [183], widely appreciated in the realization of thin-film transistors. Pentacene has limited solubility, and TIPS-pentacene is one of the most interesting derivatives, with increased solubility and processability [184]. Compatibilization of a P3HT:PC_71_BM blend with different percentages of TIPS-pentacene results in an enhancement of power-conversion efficiency up to 4.13% (with 33% increase compared to the benchmark device), by the addition of a minimum amount of CB (0.6 wt.%).

#### 2.2.2. Fulleroids

The number of examples reporting fullerene derivatives acting as compatibilizers is quite limited. Nevertheless, it is worth mentioning the work performed by Kim and collaborators, who tried to apply the same strategies we described in the previous sections concerning polymers, but on a molecular scale. In detail, a small library of fulleropyrrolidine derivatives was prepared [185]. The molecular structures, namely nT-C_60_, differ by the number of thiophene rings in the lateral chain connected to the pyrrolidine ring. Derivatives have n = 2, 4, and 8, and the authors demonstrate that the structure comprising four rings has the highest efficiency as interface modulator, compatibilizing a typical P3HT:PC_61_BM blend. Optical microscopy and SEM/EDX mapping revealed the absence of a macrophase separation within photoactive layers, with improved electrical characteristics. If 4 represents the optimum, 8T-C_60_ does not arrest macrophase separation, while 2 looks to have a too-short chain, making it unable to diminish interfacial energy as it happens for derivative 4. Later, an alcohol-soluble fullerene derivative, FN-C60, finds a somewhat different application as an interfacial layer between the active layer and Al cathode for PSCs [186]. A variety of blends, including benzothiadiazole polymers and PC_71_BM, were tested, with increased electric performances by 20−70%; results are justified on the basis of an improved mixing interface and the intrinsic elevate efficiency of electron transportation and collection operated by the fulleropyrrolidine.

**Figure 12:**
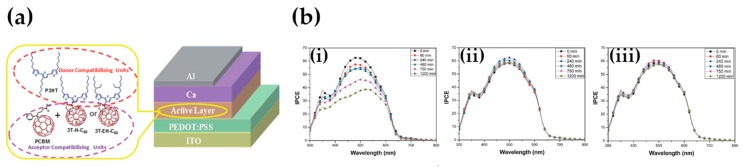
Terthiophene-C_60_ dyads compatibilizers. (**a**) Fulleropyrrolidine derivatives bearing π-conjugated terthiophene and two hexyl (H)/ ethylhexyl (EH) chains (3T-H-C_60_ and 3T-EH-C_60_) used as a CB of P3HT:PCBM active layers. (**b**) IPCE spectra of the BHJ solar cells based on (i) P3HT.PCBM, (ii) P3HT:PCBM:3T-H-C_60_ (1 wt.%), and (iii) P3HT.PCBM:3T-EH-C_60_ (3 wt.%), after annealing at 130 °C at different times (0–1200 min). Reproduced from Reference [187], with permission from the Royal Society of Chemistry.

One other interesting report came in 2015, when Raja et al. reported the synthesis of two novel fulleropyrrolidines, 3T-H-C_60_ and 3T-EH-C_60_, which are characterized by the presence of terthiophene fragments, with alkyl chains as substituents [187]. Compared with the standard PC_61_BM, tiny amounts of the CB-molecules enhance the short-circuit current density, which can be related to the formation of small PCBM clusters within the P3HT matrix; limited thermal mobility, as revealed by optical microscopy, transmission electron microscopy, and photoluminescence measurements, leads to the formation of an extremely stable morphology (even under long-term exposure to elevated temperatures) and an expected increase in PCE values (Figure 12).

#### 2.2.3. Non-Conjugated Molecules

Little heterocycles like 1,4-piperazine and 1,4-dithiane have been tested as efficient compatibilizers [114]. However, the literature is quite poor in the number of reports, to this regard. A considerable number of studies are definitely pointing their attention at 1,8-diiodooctane, aka DIO. This simple halogenoalkane cannot be properly defined as a compatibilizer, but most likely belongs to the solvent additives class. By the way, it is worth considering the opportunity to leave room for an excursus, detailing this specific molecule due to its ubiquitous presence in the literature as a benchmark molecule for compatibilizers. In other words, a new compatibilizer is worth being adopted when its presence leads to an improved OPV performance compared to the employ of a simple solvent additive, which can be lately removed; on the contrary, compatibilizers are permanent components of the blend, by definition.

DIO assumes a primary role as solvent additive, thanks to the high boiling point and compatibility with the widely established aromatic solvents [162]. Seminal reports of this interesting and multipurpose compatibilizing molecule can be found in the paper by Liang et al. [188], who systematically describe the preparation and characterization of a PTB7/PC_71_BM photovoltaic device, where the presence of DIO allows it to reach a relevant performance increase. One of the most intriguing aspects of DIO is that, although it possesses an extremely simple molecular skeleton, significant enhancement in the quality of the photoactive layer morphology can be achieved. This is due to the prevention of large PCBM domains (which negatively limit excitons dissociation) [189], which finds explanation in the strong interactions between partially negatively charged iodine substituents and electron deficiency typical of fulleroderivatives [190]. As widely reported [191], AFM images evidence smoother films and less heterogeneous surface features. The reason behind this evidence is mainly related to the efficient dissolution of PCBM aggregates, promoting nanoscale phase separation [192], and resulting in a homogeneous distribution of the molecular acceptor within the photoactive blend [190]. Consequently, the increased D/A-I area promotes a more efficient generation of charge carriers [193,194]. The dimension of PCBM nanoaggregates formed in the presence of DIO is widely documented, using several techniques, such as AFM [87] and soft X-ray scattering studies [195].

Although there is a plethora of studies where DIO is considered as benchmark molecule also for CBs, and novel potential CB-molecules are compared with it, only a few reports really show new structures which tend to an optimization of its molecular design. Among those, some interesting reports focus on the exchange of iodine-atoms with thiol-functionality [196,197].

Salim et al. presented a deep investigation of thiol-derivatives, where chain-length is related to the final device performance [196]; in particular, eight-member chains are justified to be an optimal compromise, since shorter dithiols (five-atom chains) produce poor intermolecular interactions, while longer derivatives (nine-atom chains) generate too-strong and prolonged interactions with the photoactive materials. A recent step forward comes with the detailed study by Yuan and colleagues, who compare devices produced with different photoactive materials and a triad of CB-molecules, namely DIO and another two materials (DClO and DBrO), where iodine is enhanced with chlorine or bromine atoms, respectively [163]. Results show that better performances can be obtained by using the brominated version of DIO, calling for new experiments and more studies in order to establish the new benchmark CB.

All good news up here? Yes, but employing those additives should be done in a judicious and critical way, so some drawbacks should be explored. In the general excitement, a special mention goes to Tremolet de Villers et al., who willed to shed light on some ignored aspects. In their report [198], they investigate the long-term stability of PTB7:PC_71_BM photoactive layers under ambient light illumination. They observed photodegradation phenomena, whose effects are dramatically amplified in presence of residual DIO within the photoactive layer. Using a combination of techniques, they monitored the whole phenomenon: X-ray fluorescence was employed for detecting and monitoring the quantity of residual DIO, while GIWAXS and FT-IR techniques were used for the film modification within the irradiation cycles, from both structural and chemical point of view. The authors suggest in the end that, although DIO employ is beneficial to produce OSCs, its impact on the device performance should not be considered comparable to that of compatibilizers, and its removal should be systematically monitored, once its role as morphological tuner has been accomplished. At the end of this section, it is important to state that the review could not ignore the existence of such a broadly used additive, which is generally employed as a benchmark for what we (have tried to correctly) define a compatibilizer; on the other hand, we consider it important to advertise the paper by Tremolet de Villers et al., in order to stress the fact that, although DIO provides an initial beneficial effect to BHJs, it becomes a source of instability, as it permanently remains blended within the photoactive layer.

## 3. Conclusion and Perspective

The world of BHJ-based Organic Photovoltaics has become established enough to produce valid alternatives to the traditional silicon-based technologies, at least for all those unconventional PV applications which do not allow the use of crystalline silicon, e.g., in the fields of building-integrated photovoltaics and wearable optoelectronics. Nevertheless, there is room for improvements from both the conceptual (geometry of the device and materials of choice) and the practical (large-scale fabrication and processing techniques) point of view. Compatibilizers constitute a brilliant example of how it is possible to condense several necessities into a single material. Polymeric or molecular CBs offer a valuable approach to the creation and stabilization of an efficient donor/acceptor interface, thus contributing in a beneficial way to the overall electrical performance of OPV devices. Their processing can be easily implemented for large-scale productions, paving the road for their successful implementation in mass-production products. The review revealed that significant progresses have been achieved in the past decade in the use of CBs in OPV devices, and a lot of scientific reports on this topic have been produced with the noble purpose of achieving an increased basic knowledge on the field. A large portion of the above-discussed studies was focused on the most popular D/A combination, namely P3HT:PCBM, and on the employ of compatibilizing block copolymers featuring thiophene and/or fullerenic fragments in order to maximize the miscibility with (at least one of) the blend components. In almost all the cases, favorable morphological effects induced by the CB approach were noted, comprising diminished phase separation; enhanced thermal and mechanical stability; increased crystallization of P3HT; reduced agglomeration of PCBM, etc.; and translating to higher photovoltaic performances. More recently, the CB concept has been expanded to other D/A combinations, and alternative polymeric or molecular structures with appropriate interfacial activity are continuously been developed/selected to be used as CBs toward higher efficiency and stability. Between the lines of our review, it is possible to reveal that polymer chemistry is not confined to history books, but continues to be a valid tool for tackling the issue; at the same time, more and more molecular species are evaluated, aiming at the best control of microscopical properties.

In conclusion, compatibilizers constitute a smart way to offer an alternative route to the improvement of OPVs. A simple and effective strategy is provided, which aims at boosting the performance of old fashion but still valid donor/acceptor materials, instead of pursuing the isolation of new materials with higher complexity and, in the end, higher production costs.

We hope that our review succeeded in introducing a more precise use of the terminology concerning compatibilizing materials. Our guidelines should provide the reader with a better understanding of how such polymeric/molecular components can deeply affect the performance of organic photovoltaics; moreover, we tried to offer a broad overview focused on the current trends, looking at the so-far reported structures and employed scaffolds, as well as the most important achievements in terms of the final device performance.

## Figures and Tables

**Table 1:** A summary of the polymeric CBs, including the nomenclature provided in the reference articles, the major role(s) played in the device, and the compatibilized blend. CBs are reported according to the label provided by the authors in the cited article and reported by us in Figure 4.

CBs	Definition (according to the Reference)	Major Role(s)	Compatibilized Blend
5	modulator [151]	morphology modulator	P3HT:PCBM
P3HT-*b*-PTCNE	compatibilizer [138]	morphology modulator, interface stabilizer	P3HT:PCBM
F6T2-*b*-PS(PCBM)	compatibilizer [149]	increasing operational stability	F6T2:PCBM
HOC-P3HT-COH	compatibilizer [133]	morphology stabilizer, thermal stress stabilizer	P3HT:PCBM
J71	compatibilizer [146]	morphology modulator, interface stabilizer, absorption range extender, energy levels matcher	PBDB-T:PNDI-2T-TR
P2VP	additive [155]	morphology modulator, device stabilizer	PTB7:PCBM
P3HT-(P3HT-5F)- (P3HT-5F-N-C_X_)	additive [150]	morphology modulator, absorption range extender	P3HT:PCBM
P3HT-2Py-*x*	compatibilizer [137]	morphology modulator, photovoltaic activity modulator	P3HT:PCBM
P3HT-*b*-C_60_	compatibilizer [148]	morphology modulator, thermal stress stabilizer	P3HT:PCBM
P3HT-*b*-P2VP	compatibilizer [135]	interface modulator, thermal stress stabilizer, mechanical stabilizer	P3HT:PCBM
P3HT-*b*-P2VP	additive [136]	interface modulator	P3HT:TiO2
P3HT-*b*-P3PHT	additive [139]	morphology modulator, thermal stress stabilizer	P3HT:PCBM
P3HT-*b*-PS	compatibilizer [140]	morphology modulator	P3HT:PCBM
P3HT-*b*-PS	compatibilizer [141]	morphology modulator, interface stabilizer	P3HT:PCBM
P3HT-*b*-PS	compatibilizer [142]	morphology modulator, charges mobility booster	P3HT:PCBM
P3HT-*b*-PSFu	compatibilizer [147]	interface stabilizer	P3HT:PCBM
P3HT-*b*-PTMSM	additive [131]	passivation layer precursor	P3HT:PCBM
P3HT-*b*-P4VP	structuring agent [134]	interface modulator	P3HT:PCBM
PBDB-T	(ternary cell) [145]	morphology modulator	DRCN5T:PCBM
PBDTO-T	additive/dopant [144]	morphology modulator, charges mobility booster, energy levels matcher	PBDB-T:IT-M
PCBTDPP	(ternary cell) [143]	charges mobility booster, absorption range extender	P3HT:PCBM
PFLAM	additive [152]	charges mobility booster	P3HT:PCBM
P*x*	additive [132]	decreasing the rate of recombination processes	*rr*-P3HT:PCBM
PTB7-*b*-PNDI	additive [154]	morphology modulator	PTB7:PCBM
THC8	additive [153]	morphology modulator, absorption range extender, charges mobility booster	P3HT:PCBM

**Table 2:** A summary of the small-molecule-based CBs, including the nomenclature provided in the reference articles, the major role(s) played in the device, and the compatibilized blend. CBs are reported according to the label provided by the authors in the cited article and reported by us in Figure 9.

CBs	Definition (according to the reference)	Major Role(s)	Compatibilized Blend
1-chloronaphthalene	additive [170]	morphology modulator	PTVPhI-Eh:PCBM
1-chloronaphthalene	additive [171]	morphology modulator	P3HT:PCBM
1-chloronaphthalene	additive [172]	morphology modulator	P4T2F:PCBM
1-chloronaphthalene	additive [173]	morphology modulator	PBDTTPD:PCBM
3FMBPA	additive [179]	traps passivation	(PCDTBT:PCBM):ZnO
alkylthiophenes	additive [158]	morphology modulator	P3HT:PCBM
benzenes	additive [168]	morphology modulator	P3HT:PCBM
benzenes	additive [169]	morphology modulator	PTB7-Th:PC71BM
BT	surface modifier [180]	introducing a dipole at the interface. Boosting charges mobility	(PTB7-Th:PC71BM):ZnO
diphenyl ether	additive [166]	morphology modulator	BDT-QX:PCBM
diphenyl ether, 1-chloronaphthalene	additives [164]	morphology modulator, charges mobility booster	PBDB-T:m-ITIC
diphenyl ether, 1-chloronaphthalene	additives [165]	morphology modulator, absorption range extender	PTB7:PCBM
DTBT	additive [163]	morphology modulator, charges mobility booster	PtzBI/P(NDI2OD-T2)
F-DPE	additive [167]	morphology modulator	PTB7-Th:P(NDI2OD-T2)
FN-C_60_	/ [186]	morphology modulator, charges mobility booster	P3:PCBM
hydroxypyridines	additive [159]	morphology modulator, suppression of oxygen side-effects	P3HT:PCBM
hydroxypyridines	additive [161]	morphology modulator, thermal and air stability booster	PEDOT:PSS
hydroxypyridines	additive [162]	morphology modulator	PDPP3T:PCBM
IT-4F	(third component) [157]	morphology modulator, charges mobility booster	PBTA-PS:ITIC
naphthalenes	additive [174]	morphology modulator	P3HT:PCBM
naphthalenes	additive [175]	morphology modulator	PDTSTPD:/PCBM
PBD	dopant [181]	morphology modulator, decreasing work function, increasing conductivity	(PBDB-T:IT-M):ZnO
SA-*x*	additive [178]	morphology modulator, charges mobility booster	PBDB-TF:IT-4F
SH-na	additive [176]	morphology modulator, charges mobility booster	PTB7:PCBM
TDGTPA	electron-selective interlayer [156]	energy levels matcher	P3HT:PCBM
TiPS-pentacene	dopant [182]	energy levels matcher	P3HT:PCBM
*x*T-C_60_	compatibilizer [185]	morphology modulator, interface stabilizer	P3HT:PCBM
*x*T-H-C_60_	compatibilizer [187]	morphology modulator	P3HT:PCBM
